# Editorial: *Ex vivo* Liver Machine Perfusion

**DOI:** 10.3389/fsurg.2022.861575

**Published:** 2022-03-03

**Authors:** Arash Nickkholgh, Daniel G. Maluf, Peter Schemmer

**Affiliations:** ^1^Department of General, Visceral and Transplant Surgery, Ruprecht-Karls University, Heidelberg, Germany; ^2^Program in Transplantation, University of Maryland Medical Center, University of Maryland School of Medicine, Baltimore, MD, United States; ^3^General, Visceral, and Transplant Surgery, Department of Surgery, Medical University of Graz, Graz, Austria

**Keywords:** machine perfusion, organ preservation, transplantation, liver, *ex vivo*

The success of liver transplantation (LT) as the life-saving standard of care for the irreversible liver diseases has been limited by the shortage of optimal grafts. The so-called “extended criteria donor (ECD),” including but not limited to the livers with steatosis, donors after circulatory death (DCDs), or livers from older donors, have been used to expand the donor pool. These organs are specifically more susceptible to ischemia prior to and during the static cold storage. Moreover, the exact prediction of their posttransplant function has not yet been possible.

With the introduction and recent growing clinical implementation of *ex vivo* liver machine perfusion (evLMP), the field of LT has been experiencing a paradigm shift in the preservation technology. With the more objective potential to evaluate the viability of the liver grafts (1), evLMP might eventually expand the organ pool (2), decrease the post-transplant complications (3) and thus decreasing the hospital length of stay (4). Furthermore, this technology has the potential to serve as a platform for donor preconditioning protocols (5).

The present Research Topic includes some interesting works from all around the globe on different perspectives of evLMP. Serifis et al. from Boston, USA, in their compact review have described the principles of the two major machine perfusion modalities, the hypothermic and the normothermic machine perfusion, and have summarized the clinical trials and studies concerning each modality. The authors have also described normothermic regional perfusion, as well as controlled oxygenated rewarming used to combine different machine perfusion techniques. They have also discussed the future applications of the machine perfusion.

Attard et al. from Birmingham, UK, have introduced a novel evLMP split protocol as a feasible proof-of-concept for providing comparative controls for pre-clinical normothermic machine perfusion research for cellular therapies to investigate cellular phenotype and lineage changes and future pharmacological interventions of donor liver before implantation. Haque et al. from Boston, USA, have used this protocol to recondition discarded DCD livers with tissue plasminogen activator (tPA) while on normothermic evLMP and assessed injury to peribiliary vascular plexus and mural stroma.

Tingle et al. from Newcastle, UK, have reported two cases of methaemoglobinaeima as a complication of normothermic evLMP associated with large reductions in oxygen delivery and oxygen extraction.

Cheng et al. from Zhengzhou University in China have administered an exogenous Activating Transcription Factor 6 activator with evLMP and explored its protective effects in a DCD rat liver model, including, but not limited to, reduction in sinusoidal injury scores, changes of ATP level in the liver, and expression of cytochrome c in mitochondria.

The evLMP technology is very promising and has already paved its way into routine clinical implementation in many transplant programs. However, many questions must be answered, and many challenges must be addressed before broader utilization. Examples are the definition of ideal end points for the clinical trials, valid markers for the prediction of viability and post-transplant outcomes, the best approach toward different clinical scenarios, the cost, and the potentials and applications for graft protective strategies and cell therapy. Examples of the latter are *ex vivo* interventions targeting the ischemic injury, and the implementati in CRISPR technology to induce tolerance (6). With the remarkable ongoing research and evolving clinical trials in the field, many of these questions will be hopefully answered soon.

## Author Contributions

AN drafted the manuscript. PS and DM revised and gave approval for publication of this manuscript. All authors contributed to the article and approved the submitted version.

## Conflict of Interest

The authors declare that the research was conducted in the absence of any commercial or financial relationships that could be construed as a potential conflict of interest.

## Publisher's Note

All claims expressed in this article are solely those of the authors and do not necessarily represent those of their affiliated organizations, or those of the publisher, the editors and the reviewers. Any product that may be evaluated in this article, or claim that may be made by its manufacturer, is not guaranteed or endorsed by the publisher.
